# Modulation of feedback-related negativity during trial-and-error exploration and encoding of behavioral shifts

**DOI:** 10.3389/fnins.2013.00209

**Published:** 2013-11-14

**Authors:** Jérôme Sallet, Nathalie Camille, Emmanuel Procyk

**Affiliations:** ^1^INSERM U846, Stem Cell and Brain Research InstituteBron, France; ^2^Université Lyon 1, Université de LyonLyon, France; ^3^Decision and Action Laboratory, Department of Experimental Psychology, University of OxfordOxford, UK; ^4^Institut des Sciences Cognitives, Centre National de la Recherche ScientifiqueBron, France

**Keywords:** cingulate cortex, reward prediction error, feedback-related negativity, trial and error exploration

## Abstract

The feedback-related negativity (FRN) is a mid-frontal event-related potential (ERP) recorded in various cognitive tasks and associated with the onset of sensory feedback signaling decision outcome. Some properties of the FRN are still debated, notably its sensitivity to positive and negative reward prediction error (RPE)—i.e., the discrepancy between the expectation and the actual occurrence of a particular feedback,—and its role in triggering the post-feedback adjustment. In the present study we tested whether the FRN is modulated by both positive and negative RPE. We also tested whether an instruction cue indicating the need for behavioral adjustment elicited the FRN. We asked 12 human subjects to perform a problem-solving task where they had to search by trial and error which of five visual targets, presented on a screen, was associated with a correct feedback. After exploration and discovery of the correct target, subjects could repeat their correct choice until the onset of a visual signal to change (SC) indicative of a new search. Analyses showed that the FRN was modulated by both negative and positive prediction error (RPE). Finally, we found that the SC elicited an FRN-like potential on the frontal midline electrodes that was not modulated by the probability of that event. Collectively, these results suggest the FRN may reflect a mechanism that evaluates any event (outcome, instruction cue) signaling the need to engage adaptive actions.

## Introduction

The evaluation and utilization of outcomes are crucial for the exploration and exploitation of resources available in the environment. The feedback-related negativity (FRN), a mid-frontal event-related potential that is elicited in various cognitive tasks at the onset of sensory feedback signaling an outcome, is widely used to study error and reward-related processes [for review see San Martin (2012); Walsh and Anderson (2012)]. Functional MRI (fMRI) studies and electroencephalography (EEG) source reconstructions notably implicate the anterior part of the cingulate cortex as a potential source for the FRN (Ullsperger and Von Cramon, 2001; Gehring and Willoughby, 2002; Holroyd et al., 2004; Amiez et al., 2012, 2013). The aforementioned region is likely to correspond to the anterior midcingulate cortex (aMCC) and might extend to the perigenual ACC (pACC) according to Vogt's subdivisions of the cingulate cortex (Vogt, 2009a,b). Here we refer to the cingulate region that encodes feedback as aMCC.

Several models have been proposed to explain the role of the aMCC in outcome processing (Holroyd and Coles, 2002; Alexander and Brown, 2011; Khamassi et al., 2013). The initial, and still influential, Holroyd and Coles Reinforcement Learning-ERN model proposed that through the direct meso-cortical dopaminergic pathway a negative prediction error-signal disinhibits aMCC neurons, which thereby produce the cortical error signal (Holroyd and Coles, 2002). Some aspects of this theory have been challenged. In particular whether the aMCC outcome related response is specific or even relates to error processing (Williams et al., 2004; Cohen et al., 2007; Oliveira et al., 2007; Sallet et al., 2007; Quilodran et al., 2008; Kennerley and Wallis, 2009; Vezoli and Procyk, 2009; Hayden and Platt, 2010; San Martin et al., 2010; Amiez et al., 2012; Walsh and Anderson, 2012) and whether or not a reward prediction error (RPE) is encoded in the aMCC (Holroyd et al., 2003; Ito et al., 2003; Yasuda et al., 2004; Amiez et al., 2005, 2012; Haruno and Kawato, 2006; Bellebaum and Daum, 2008; Quilodran et al., 2008; Bellebaum et al., 2010; Cavanagh et al., 2010, 2012; Rutledge et al., 2010; Sailer et al., 2010; Chase et al., 2011; Hayden et al., 2011a; Kennerley et al., 2011; Pfabigan et al., 2011; Talmi et al., 2013). Recent interpretations suggest that the aMCC feedback-related activity is not only involved in processing negative outcomes, but instead reflects a mechanism that evaluates outcomes and the associated need to engage different adaptive actions (Cohen et al., 2011; Amiez et al., 2012; Karlsson et al., 2012; Rushworth et al., 2012).

As mentioned above the sensitivity of the FRN to both positive and negative RPE is still debated [for review see San Martin (2012)]. The first goal of our experiment is therefore, to address this question of sensitivity of the FRN to RPE. To contrast with most of the previous experimental designs developed to study FRN properties, we did not use a two choice task in which subjects were asked to learn, or to guess, the correct answer. We adapted a multiple choice task that we previously used in our studies in humans and monkeys (Quilodran et al., 2008; Amiez et al., 2012). The task (problem-solving task: PST) is a 5 choice task with two distinct alternating periods: an exploration period during which the subject searches by trial and error for the correct response, and an exploitation period during which the subjects were allowed to repeat the rewarded response. A visual signal, called “signal to change” (SC) indicated (1) the end of the exploitation period, and (2) that a new problem had to be solved. Two critical task features are: (i) each feedback signaling an error has an impact on the decision to be made in the next trial, thus, there is a clear link between trials, (ii) because the trial and error process eliminates solutions one by one, the probability of finding the correct target, and thus, the expectation, increases naturally during the search process. The design of our task ensured that participants would not only focus on the correct choice but also monitor incorrect choices in order to solve a problem without making perseverative error. Indeed our fMRI version of the task showed increased activity at the time of the feedback, not only in the cingulate cortex but also in the frontopolar cortex (Amiez et al., 2012), a region involved in encoding information about alternative courses of action (Rushworth et al., 2012). We based our experiment on the idea that the properties of a signal devoted to reinforcement learning should be optimally expressed and modulated in a situation in which learning occurs, and in which subjects are involved in active adaptation. We thus, hypothesized that the FRN could be modulated by both positive and negative prediction errors within a single experimental context, if it necessitates the monitoring of both correct and incorrect choices. Moreover the use of the PST would enable direct comparison of the results with those of our previous studies in humans and monkeys (Quilodran et al., 2008; Amiez et al., 2012).

Finally it has been shown that aMCC cells encode behavioral transitions between exploration and exploitation (Procyk et al., 2000; Amiez et al., 2005; Quilodran et al., 2008). In the PST, the transition between exploitation and a new exploration period is indicated by a visual stimuli and aMCC cells code for these events (Amiez et al., 2005). It has also been shown that an FRN could be recorded following a cue that indicates a future outcome (Holroyd et al., 2011). Thus, we hypothesized that an FRN could be recorded not only following the occurrence of feedback, but also following the cue indicating the need to switch from exploitation to exploration.

## Materials and methods

### Subjects

The study was approved by the local ethical committee (Lyon-A) and conducted according to the French law for biomedical research. Prior to the study, subjects were briefed on the nature of the experiment and given standardized written task instructions. Subjects gave their written informed consent. 12 males subjects were analyzed for this study. Subjects were all right-handed, free of medication and without any neurological disorder. All subjects had received more than 13 years of education (17.62 ± 1.80), had normal or corrected-to-normal vision. They were on average 24.15 ± 2.48 years old. Subjects were comfortably seated at 90 cm in front of a 17-inch video monitor, on which visual targets were presented using EPrime 1.1 (Psychology Software Tools, Pittsburgh, PA, USA). Responses were made by moving a cursor (a white cross) on the screen with a computer mouse.

### Behavioral task (figure 1a)

During the problem solving task subjects were asked to find, by trial and error, the correct target among 5 potential targets presented simultaneously. A trial started with the appearance of a white central fixation point for 1500 ms before the onset of the 5 targets. Targets were 1 cm diameter discs equally distributed on a 5 cm radius circle. Subjects were instructed to fixate the fixation point during the entire trial. 1000 ms after target onset, the mouse cursor (i.e., a white cross) appeared over the fixation point. The subject could then respond by moving the cursor toward one of the targets. All 5 targets were switched off when the cursor reached a virtual response field (twice the target size) defined around the target chosen by the subject. Following a 1500 ms delay the feedback stimulus was presented for 800 ms. A 2100 ms inter-trial interval (ITI) preceded the onset of the next trial. Respectively, correct and incorrect feedback consisted of a central green or red square (6.5 * 5.9 cm) displayed at the center of the screen. After an incorrect choice, the subject had to continue his search for the correct target. The discovery of the correct target was thus, indicated by the first green feedback, which ended the search period. The subject was asked to repeat his choice for 0 to 2 trials. This second period of the task constitutes the repetition period. During this repetition of correct choice phase the different lengths of repetition period (0, 1, or 2 trials long) were equally represented. The repetition phase ended 800 ms after feedback offset with a blue ellipse appearing at the center of the screen for 1000 ms. This “signal to change” (SC) cue, indicated the start of a new problem and thus, the initiation of a new search. Because the FRN is not sensitive to the physical properties of the eliciting stimuli (Holroyd and Coles, 2002), the feedback stimuli were not counterbalanced across subjects. Feedback was not associated with monetary gain or losses. In 31% of trials a meaningless yellow rectangle appeared for 600 ms, 400 ms after the SC offset, or 1500 ms after the feedback offset. Unfortunately however, too many recordings were contaminated by eye blinks and as a result we were unable to include the signal associated with the meaningless yellow rectangle in our analysis.

### EEG recordings and analyses

The experiment was conducted in an unshielded EEG laboratory at the Cognitive Science Institute, Lyon, France. Scalp voltages were collected with a 65-channel Geodesic Sensor Net and amplified with an AC coupled, 65-channel, high input impedance amplifier (200MÙ, Net Amps, Electrical Geodesics Inc., Eugene, OR, USA). Amplified voltages (0.1–200 Hz band pass) were sampled at 500 Hz. Individual electrodes were adjusted until the measured impedance stayed <50 kΩ. The experiment was divided in three sessions (150 ± 10 trials per session). Between each session, impedance was checked and readjusted when necessary. The use of a relatively high impedance threshold prevented us from doing a time-frequency analysis.

EEG analyses were performed with in-house scripts and processing pipelines (Matlab 7.0, The MathWorks Inc. Natick, MA, USA). These routines searched for abrupt changes in signal voltage, indicative of artifacts. Trials contaminated by eye movements, eye blinks or abnormal changes in electrode voltage (>100 μV) were removed prior to any analysis. On average the artifact rejection procedure eliminated 10% of trials per subject.

The signal was re-referenced using the right mastoid electrode. ERP were calculated by averaging signals recorded between −200 to +1000 ms from feedback or SC onset. A baseline correction was applied by subtracting the average value of the 200 ms period that preceded the display of the feedback or the SC. Our analyses were confined to the 800 ms following the presentation of the feedback or the SC.

Based on previous published studies (Yeung and Sanfey, 2004; San Martin et al., 2010; Pfabigan et al., 2011; Cavanagh et al., 2012; Ferdinand et al., 2012), we focus our report on data obtained from a frontal electrode (FCz) for our analysis on the FRN, and from a parietal electrode (PCz) for our analysis on the P300. The FRN was quantified by the difference between the most negative peak arising between 200 and 350 ms after feedback onset and the average voltage of the immediately preceding and following positive peaks (Yeung and Sanfey, 2004). We considered the P170 as the most positive peak in the time period 150–250 ms after feedback onset and the P300 as the most positive peak in the time period 250–600 ms after feedback onset. All measurements were taken on the averaged ERP waveforms. FRN and P300 latencies were assessed at the time of their peaks.

**Figure 1:**
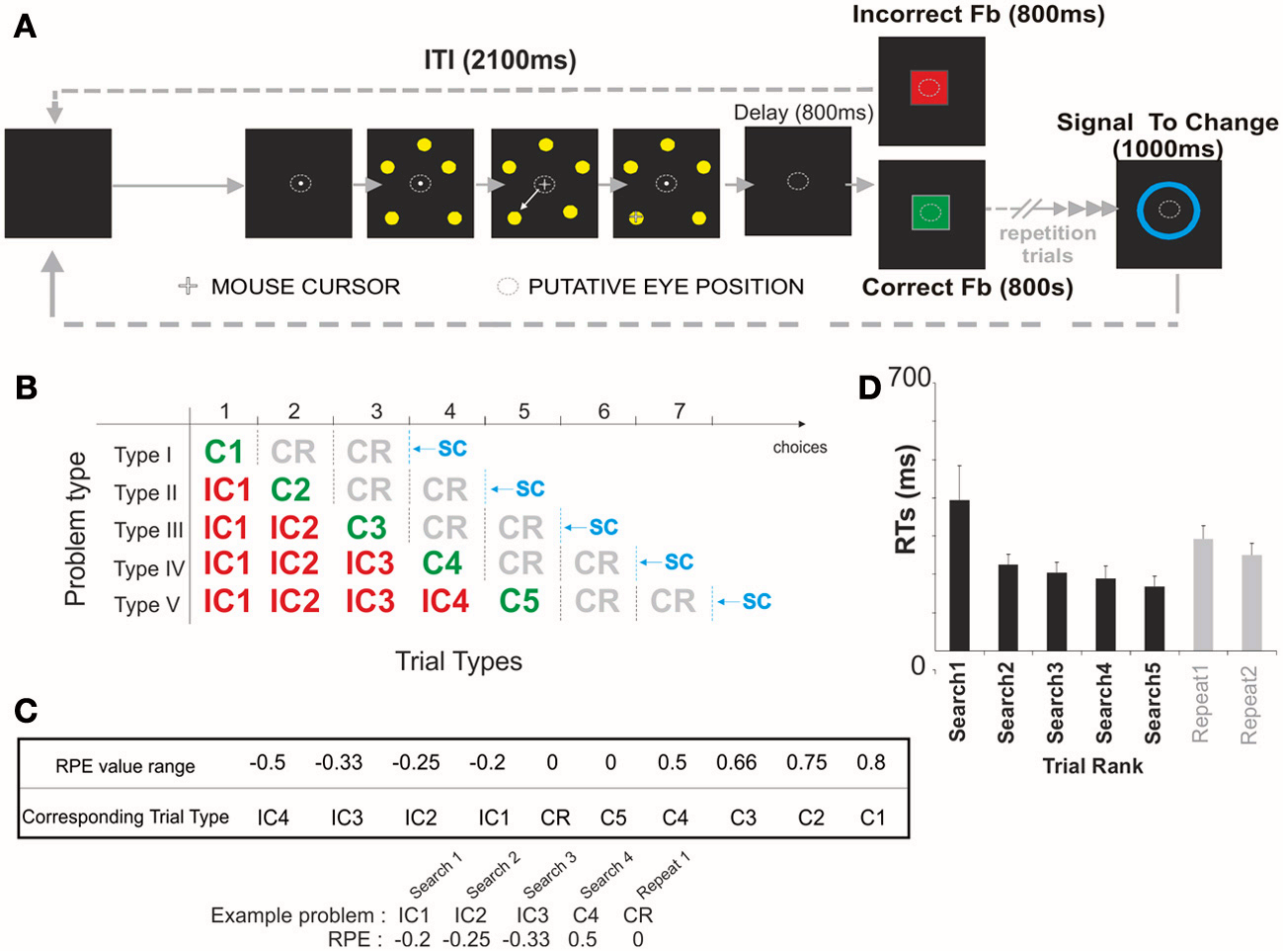
**Behavioral task and performance. (A)** Schematic representation of the Problem Solving Task. The goal of the subject was to find by trial and error which one of 5 stimuli was associated with positive feedback. Each trial started with the target presentation. During this period subjects had to chose one stimulus using four computer mouse buttons. Once a target was selected using a computer mouse, the 5 stimuli were switched off. A 800 ms delay followed and preceded the appearance of the feedback. The feedback (a positive feedback was a green square; a negative feedback was a red square) was displayed on the screen during 800 ms and its offset was followed by a 2100 ms inter-trial interval (ITI). After an incorrect choice, subjects continued to search for the correct stimulus while keeping in memory the previous erroneous choices. Discovering the correct stimulus (i.e., associated with green square feedback) ended the search period. The subjects then repeated the correct choice (i.e., repetition period, see shaded area). Immediately after the discovery of the correct target, or after one or two repetition trials, a blue ellipse appeared on the center of the screen 800 ms after the offset of the correct feedback. This ellipse presented for 1000 ms indicated that a new problem would start. **(B)** Problem types and trials. The problems the subjects were resolving were of five different types (I, II, III, IV, V). Each problem could be decomposed into up to 10 types of trial (IC1, IC2, IC3, IC4, C1, C2, C3, C4, C5, CR). The different trial types were defined based on the obtained feedback and its position within a sequence of feedback obtained during the resolution of one of the 5 types of problems. Feedback obtained at the end of trials were labeled “C” for Correct and “IC” for Incorrect; numbers following “IC” and “C” indicate the rank of the feedback in the search period. For example IC3 indicates that this is the third error of the subject in the search period. C2 indicates a correct feedback obtained after the second choice in the search period. “C” followed by “R” indicates correct trials in repetition periods. The dashed line indicates that the fact that subject is asked to repeat his choice for a variable repetition period of 0 to 2 trials. The vertical blue dashed line represent trials for which SC occurred after two repetition trials. We only represented those trial types on this figure. Vertical black dashed lines illustrate the other putative position of the SC. **(C)** Reward Prediction Error (RPE) values associated with each trial type. **(D)** Reaction Times (RTs) measured for the different trial ranks i.e., from 1 to 5 trials in the search period, 1 or 2 trials in the repetition period. All problem types were collapsed for this analysis.

To address the question of ERPs' sensitivity to feedback expectation, we separated feedback depending on their valence (correct or incorrect) and rank position in a problem. Feedback was labeled “C” for Correct and “IC” for Incorrect; numbers following “IC” and “C” indicate the rank of the feedback in a search period. For instance IC3 indicates that this is the third trial and also the third error of the subject in a search period. “C” followed by “R” indicates a correct feedback in the repetition period. Examples of possible lists of trials in a problem are shown in Figure 1B. Due to the experimental design some types of feedback were infrequent. C1, C2, C3, C4, and C5 ERPs were based—after removing artifacts- on an average of 19.47 ± 0.57 trials. The different incorrect feedback ERPs tended to be based on more trials with the exception of IC4 ERPs (IC4 = 18.86 ± 0.77; IC3 = 38.77 ± 1.4; IC2 = 55.15 ± 2.63; IC1 = 72.67 ± 3.45).

Negative and positive prediction errors (RPE) were calculated during the search period for each type of incorrect (IC1, IC2, IC3, IC4) and correct trial (C1, C2, C3, C4, C5) in the following way:

$$\text{RPE}=\left[r_{\text{obt}}\right]-\left[r_{\text{exp}}\cdot p_{\text{cor}}\right]$$

where *r*_obt_ is the obtained outcome, *r*_exp_ is the expected outcome (=1) and *p*_cor_ is the probability to be correct.

The formula was based on the following assumptions:

1. The subject is always aiming for a positive outcome.
2. The value of the obtained outcome is “0” or “1” for negative and positive outcomes, respectively.
3. The probabilities of being correct for the different trials of a search period were calculated based on the number of alternatives. The more options available, the lower the probability of discovering the correct target. For instance, for the first trial, 5 alternatives are available, so the probability of being correct is *p* = 1/5 = 0.2; for the second trial, the probability is *p* = 0.25 (4 alternatives), for the third trial, *p* = 0.33 (3 alternatives); for the fourth trial, *p* = 0.5 (2 alternatives); and for the fifth trial *p* = 1 (1 alternative). The RPE values related to each trial are represented in Figure 1C.

Statistical analyses were carried out with a significance threshold of *p* = 0.05 using Matlab scripts (Matlab 7.0, The MathWorks Inc. Natick, MA, USA) and SPSS software (IBM Corp. Armonk, NY, USA). Sphericity was tested prior to running a repeated measures ANOVA using a Maughly's test of sphericity. If sphericity was violated then a Greenhouse-Geisser correction was applied and corrected values were reported (corrected *F* and *p* values as well as epsilon value used to adjust the degrees of freedom are reported).

## Results

### Behavioral results

Each participant resolved approximately 110 problems (113.6 ± 1.4 problems). Subjects identified the correct target in an average of 2.97 ± 0.03 choices. They made very few perseverative errors (2.4 ± 0.6) across the entire session implying that the subjects had understood the task instructions. For the subsequent Reaction Time (RTs) and EEG data analyses, only correctly solved problems (i.e., problems in which the subjects did not make perseverative errors) were included. For the RTs analysis, trials of correctly solved problems were sorted according to their ranks (Search1 to 5, and Repetition trial 1 and 2), not according to the feedbacks obtained at the end of each trial (see Figure 1B). In other words, subject's reaction times IC1 and C1 trials (Search 1) were pooled because both trials correspond to the 1st choice of a search period. Similarly, IC2 and C2 trials (Search 2), IC3 and C3 (Search 3) trials, IC4 and C4 (Search 4) were pooled. Finally, C5 (Search 5), 1st trial of the repetition period (Repeat 1) and the 2nd trial of the repetition period (Repeat 2) were kept separately. The subjects' results revealed that RTs were longer at the beginning of search periods [ANOVA, *F*_(6, 66)_ = 5.49, *p* = 0.0001; see Figure 1D]. The decrease in RTs over the search period could be related to an increased expectation in obtaining the correct feedback. Similar phenomenon has been observed in our previous study in humans using a similar paradigm (Amiez et al., 2012).

### Electrophysiological results

**Figure 2:**
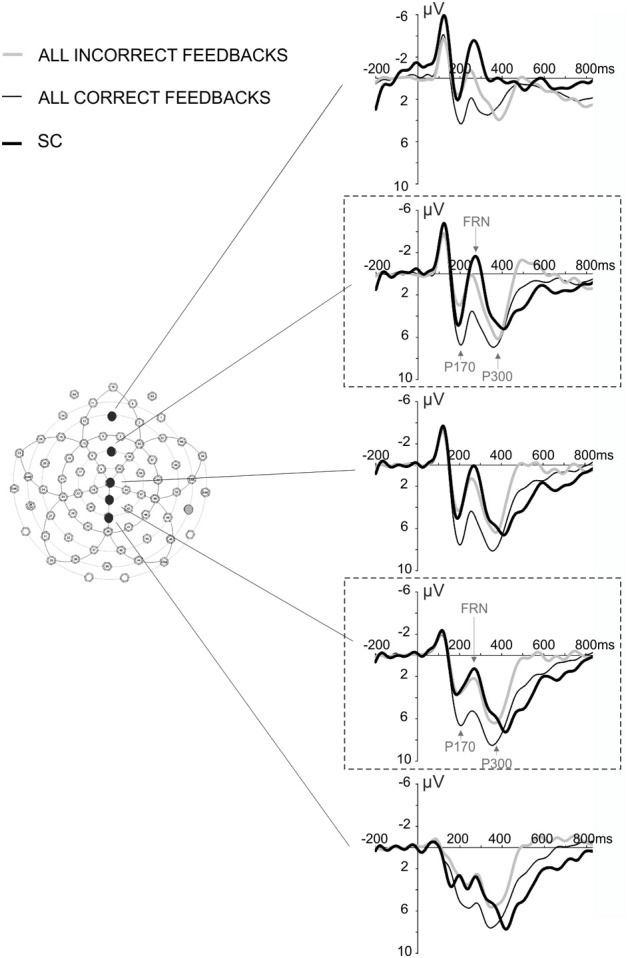
**ERPs elicited by all correct, all incorrect feedback, and SC at midline electrodes Fz, FCz, Cz, PCz, and Pz.** The dashed line frames indicate the electrode of interest, FCz and PCz. Arrows indicate the peak of the three potentials (P170, FRN, and P300) that were considered in our analysis.

We focused our analysis on two ERPs: the FRN and the P300. Based on published studies (Yeung and Sanfey, 2004; San Martin et al., 2010; Pfabigan et al., 2011; Cavanagh et al., 2012; Ferdinand et al., 2012), we focused our analyses on electrodes of interest, FCz, and PCz (Figure 2), to study the sensitivity of the FRN and the P300 to feedback valence and feedback expectation. Finally we investigated whether or not FRN could be elicited after cue indicative of behavioral shift.

### Modulations during trial and error

**Figure 3:**
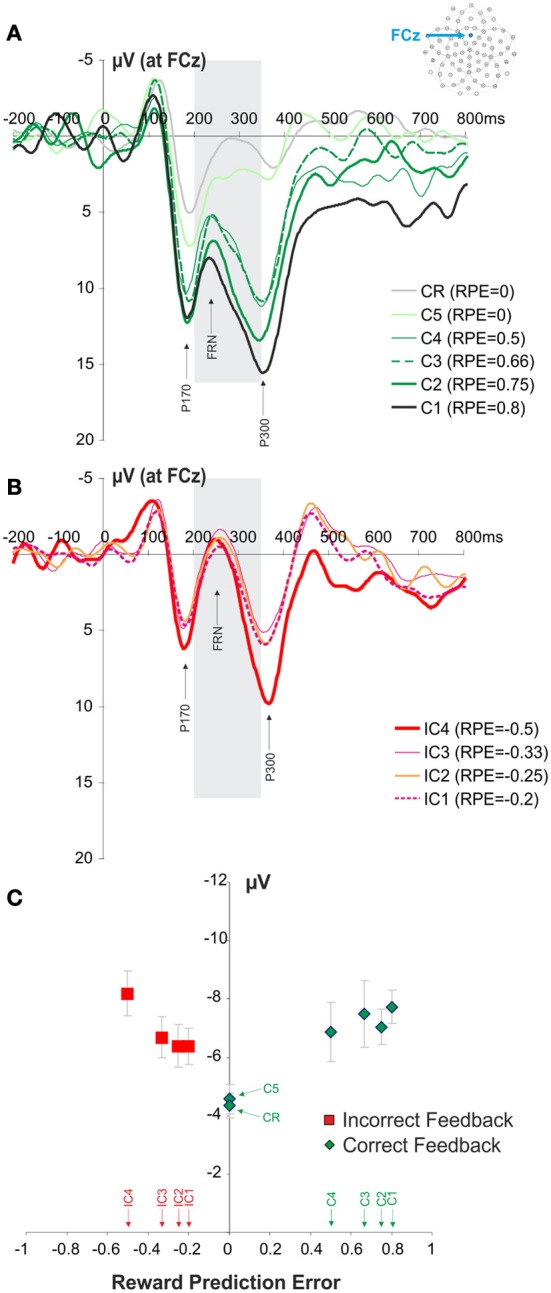
**Waveforms recorded at FCz at the presentation of the feedback and FRN modulation. (A,B)** Average waveforms elicited by the different correct and incorrect feedbacks. The panel **(A)** illustrates the signals recorded for the different positive feedbacks (C1, C2, C3, C4, C5, CR). Similarly the panel **(B)** illustrates the signals recorded for the different negative feedbacks (IC1, IC2, IC3, IC4). The gray box is indicating the time windows of interest in which we measured the amplitude of FRN (+200 to +350 ms post feedback). **(C)** Modulation of FRN amplitude according to RPE quantified at FCz. Red and green symbols represent RPE values associated with negative and positive feedbacks, respectively. Feedback types corresponding to the different RPE values are indicated above the horizontal axis.

Separating the 10 different types of feedbacks (IC1, IC2, IC3, IC4, C1, C2, C3, C4, C5, CR) revealed a strong effect of the RPE on the amplitude of the FRN (Figure 3). We found that the FRN amplitude was modulated by the RPE (see Materials and Methods) [repeated measures ANOVA, *F*_(9, 99)_ = 5.43, ε = 0.368, *p* = 0.003]. The higher the RPE value, the higher the amplitude of the FRN. During search periods the expectation of success (correct feedback) increases with the number of trials performed. In other words the certainty to find the correct response increases with the number of errors made. A complementary linear regression analysis revealed a modulation of FRN amplitude for both positive [*R*^2^ = 0.217, *F* = 19.484, *p* < 0.0001] and negative RPE values [*R*^2^ = 0.087, *F* = 4.383, *p* = 0.041]. At the fourth choice, if the correct target had not been previously discovered, the subjects were facing a two choice option. Therefore, at this stage only, the probability of the correct and the incorrect feedback are identical (see Figure 1). We used this condition in order to assess the sensitivity of FRN to feedback valence. The comparison between the correct and the incorrect feedback obtained at this search period revealed no difference in the FRN between correct and incorrect feedback (paired *t*-test, *t* = 1.4163, *p* = 0.1844). On the following fifth trial subjects only remaining option is the correct target (if the correct target had not been discovered previously). Despite being a feedback from the search period, the amplitude of the FRN for the C5 feedback was not different from the amplitude of a CR feedback (paired *t*-test, *t* = −0.4991, *p* = 0.6275).

**Figure 4:**
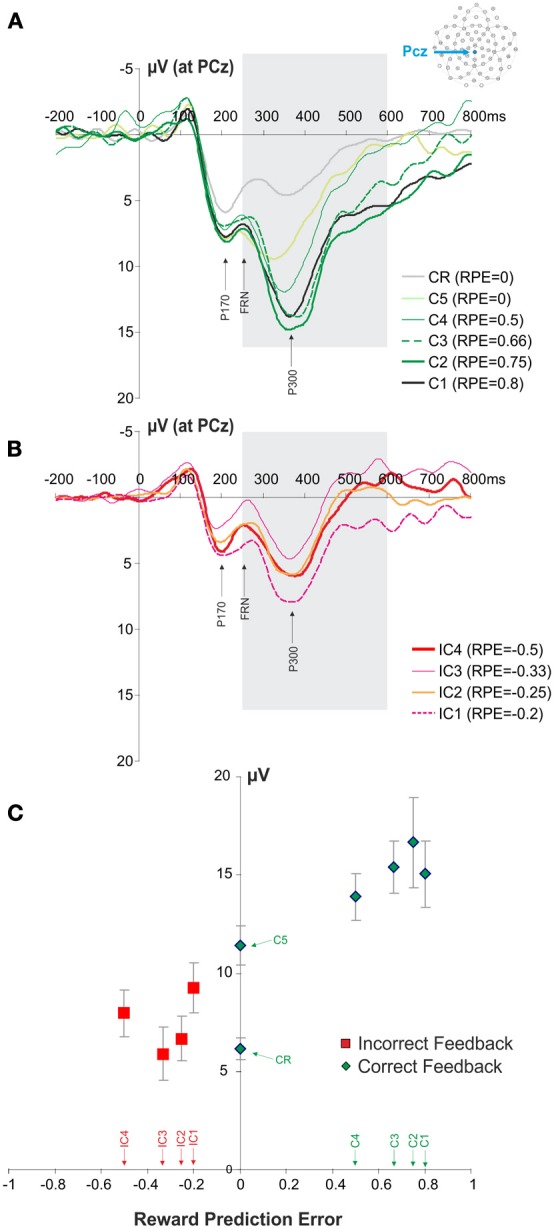
**ERPs recorded at PCz at the presentation of the feedback and P300 modulation. (A,B)** Average ERPs elicited by the different correct and incorrect feedbacks. The panel **(A)** illustrates the signals recorded for the different positive feedbacks (C1, C2, C3, C4, C5, CR). Similarly the panel **(B)** illustrates the signals recorded for the different negative feedbacks (IC1, IC2, IC3, IC4). The gray box is indicating the time windows of interest in which we measured the amplitude of P300 (+200 to +350 ms post feedback). **(C)** Modulation of P300 amplitude according to RPE quantified at PCz. Red and green symbols represent RPE values associated with negative and positive feedbacks, respectively. Feedback types corresponding to the different RPE values are indicated above the horizontal axis.

The FRN and P300 ERP components overlap in time. We therefore, performed an analysis of P300 modulation in order to verify that the observed FRN results were not in fact induced by the modulation of the P300. We hypothesized that the difference in sensitivity to outcome properties between P300 and FRN would suggest that FRN results were not induced by an overlap with the P300. The analysis revealed that the P300 amplitude also varied with the feedback types (IC1, IC2, IC3, IC4, C1, C2, C3, C4, C5, CR), i.e., with RPE values [repeated measures ANOVA, *F*_(9, 99)_ = 9.24, *p* < 0.001; Figure 4]. However, in contrast to the FRN, a linear regression analysis revealed that this effect relied on positive RPE values (*R*^2^ = 0.267, *F* = 25.589, *p* < 0.0001) and not negative RPE values (*R*^2^ = 0.002, *F* = 0.009, *p* = 0.762). In contrast with the results obtained for the FRN, the P300 was modulated by feedback valence (correct and incorrect feedback: paired *t*-test, *t* = 4.0365, *p* = 0.002). The amplitude of the P300 for C5 feedback was different from the amplitude for CR feedback (paired *t*-test, *t* = 7.4630, *p* < 0.001).

### FRN is elicited by cue indicative of behavioral shift

**Figure 5:**
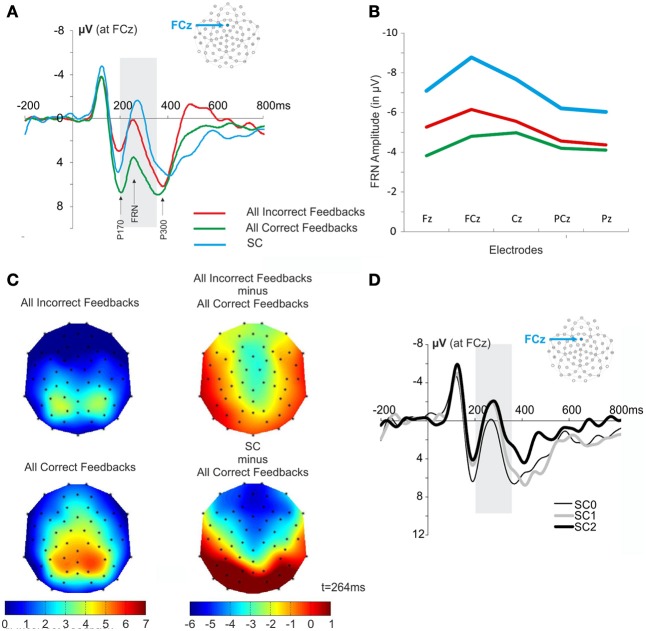
**FRN and scalp topography at the presentation of the SC and the feedback. (A)** Waveforms elicited by all incorrect feedback, all correct feedback and by SC at FCz. The gray box is indicating the time windows of interest in which we measured the amplitude of FRN (+200 to +350 ms post feedback). **(B)** Amplitude of the FRN after correct feedback, incorrect feedback and SC measured at electrode Fz, FCz, Cz, PCz, and Pz. **(C)** Scalp topography obtained at the time of average FRN latency for the different feedbacks (*t* = 264 ms). Note that the FRN latencies for all correct and incorrect feedbacks were on average 268 ms and 260 ms, respectively. **(D)** Average ERPs elicited by the different SC. The figure illustrates the signals recorded for SC elicited after the correct feedback that end the search period (SC0), after the 1st correct feedback of the repetition period (SC1), and after the 2nd correct feedback of the repetition period (SC2). The gray box is indicating the time windows of interest in which we measured the amplitude of FRN (+200 to +350 ms post SC).

The SC informed the subject that the repetition phase was over and another search was about to start. This signal elicited an ERP similar to the FRN (Figures 2, 5A). A Two-Way repeated measures ANOVA [Electrodes locations (Fz, FCz, Cz, PCz, Pz) × Feedback type (All Correct, All Incorrect, SC)] revealed an effect of electrode location on the FRN amplitude [*F*_(4, 44)_ = 5.295, ε = 0.466, *p* = 0.015] and did not reveal an interaction effect [*F*_(8, 88)_ = 2.221, ε = 0.348, *p* = 0.11]. The amplitude of the FRN tended to be maximum at the FCz, Cz electrodes (Figure 5B). The voltage cartography for the contrast between negative feedback and all first correct feedback, and between SC and all first correct outcomes, revealed comparable topography for incorrect feedback and SC (Figure 5C). Finally Two-Way repeated measures ANOVA [Electrodes locations (Fz, FCz, Cz, PCz, Pz) × Feedback type (All Correct, All Incorrect, SC)] revealed a main effect of feedback type on FRN amplitude [*F*_(2, 22)_ = 22.413, ε = 0.603, *p* < 0.001]. This result is difficult to interpret, as the probability of occurrence of the different feedbacks was different. Therefore, we did not discuss this result further.

The fact that SC appeared immediately upon discovery of the correct target, or after one or two repeats allowed us to test whether the FRN was modulated by the SC likelihood (Figure 5D). While the initial number of trials in each repetition period condition (0, 1, or 2 trials) was identical after artifact rejection, the analysis was based on a small number of trials per condition (26.2 ± 8.2). Note that one subject was excluded from this analysis because the subject made too many eye blinks at the SC. This analysis revealed no modulation of the likelihood of SC occurrence ANOVA [*F*_(2, 33)_ = 0.91, *p* = 0.4125].

In conclusion, the FRN was modulated by positive and negative prediction error but was not sensitive to the feedback valence. Furthermore, an FRN-like response was also evoked by a signal indicating the need to change response or engage in search. However, this FRN-like response was not modulated by the probability of the SC occurrence. Finally we observed a modulation of the P300 that was distinct from the effect observed for the FRN. Critically, the P300 was modulated by feedback valence and positive prediction error only.

## Discussion

Despite a relatively small number of subjects and of trials for some experimental conditions, our analysis show that during trial and error learning the FRN reflects the evaluation of both incorrect and correct feedback. We observed a modulation of the FRN by the level of expectation for successes and errors that we relates to the necessity of monitoring both correct and incorrect choices in our 5 choice problem solving task. In addition we also recorded a FRN-like potential following a SC cue. This latter result suggests that the FRN is elicited after any event relevant to behavioral adaptation and not only after feedback. However, the FRN elicited by the SC was not modulated by the likelihood of the SC to occur.

### An FRN is elicited by both incorrect and correct feedback

First, our findings confirm the presence of an FRN even after a correct feedback (Oliveira et al., 2007; San Martin et al., 2010; Chase et al., 2011; Cohen et al., 2011; Talmi et al., 2013). Discrepancies with earlier results, suggesting specificity of FRN/ERN for negative feedback, could be related to the experimental designs. In the present experimental design, in contrast with some earlier studies, subjects were required to learn from their obtained outcomes. The requirement to choose between multiple options, in the context where decision outcomes are salient, has been shown to impact feedback-related signals for learning optimal strategies (Holroyd et al., 2009; Sailer et al., 2010; Chase et al., 2011; Cohen et al., 2011; Peterson et al., 2011; Van Der Helden and Boksem, 2012; Walsh and Anderson, 2012). The FRN recorded following positive feedback could be related to the reward related properties of the aMCC. Indeed some dorsal cingulate neurons are active when the expected reward is not obtained (Shima and Tanji, 1998; Ito et al., 2003; Amiez et al., 2005; Nakamura et al., 2005; Sallet et al., 2007; Quilodran et al., 2008; Seo and Lee, 2009), but this region also contains cells that are related to the obtained reward (Williams et al., 2004; Amiez et al., 2006; Matsumoto et al., 2007; Sallet et al., 2007; Quilodran et al., 2008; Kennerley and Wallis, 2009). Finally, our results are in line with our recent fMRI data which show an increased midcingulate BOLD signal after positive and negative feedback in the search period of the PST (Amiez et al., 2012).

### FRN is coding for both positive and negative reward prediction error

In reinforcement learning the key event for successful behavioral adaption is the reward. Indeed, the RPE a parameter that seems to be critical in adaptive systems (Glimcher, 2011) is based on the calculation of discrepancy between expectation of a positive outcome and actual outcome (i.e., the reward). It is therefore, perhaps not surprising that the main signal that emerges from the reinforcement learning apparatus is coding for unexpected positive feedback. Importantly the ERN and FRN in humans seem sensitive to several pathological conditions and are altered by pharmacological treatments, in particular those involving the dopaminergic transmission (Falkenstein et al., 2001; Johansen and Fields, 2004; Zirnheld et al., 2004; Beste et al., 2006; De Bruijn et al., 2006; Vezoli and Procyk, 2009). Previous studies have attempted to determine the relationship between dorsal cingulate activity and the RPE. Contradicting conclusions have been reached from both electrophysiological works in monkeys (Ito et al., 2003; Amiez et al., 2005; Matsumoto et al., 2007; Quilodran et al., 2008; Vezoli and Procyk, 2009; Hayden et al., 2011a; Kennerley et al., 2011) and humans (Holroyd et al., 2003; Yasuda et al., 2004; Bellebaum and Daum, 2008; Bellebaum et al., 2010; Cavanagh et al., 2010; Chase et al., 2011; Pfabigan et al., 2011; San Martin, 2012; Talmi et al., 2013) regarding whether and how aMCC signals are modulated by the RPE.

Our data reveal that the FRN is modulated by both positive and negative RPE, an effect predicted by the Alexander and Brown's model (Alexander and Brown, 2011), which suggests that the medial prefrontal cortex encodes discrepancies between expected and obtained outcomes regardless of the valence. Furthermore, the lack of FRN modulation between C4 and IC4 feedback confirms that FRN is more sensitive to outcome probability than to outcome valence as also suggested by the Alexander and Brown's model and as observed by Ferdinand et al. (2012). In sharp contrast, the P300 showed a modulation by positive RPEs only. Altogether the different effects we observed for the FRN and P300 suggest that the FRN modulation by both positive and negative RPE were not driven by the overlap between the two ERPs. It would be advantageous to conduct a follow-up experiment with increased power by utilizing more subjects, additional trials per condition and a lower impedance threshold, in order to further compare the properties of the FRN and the P300.

Differences in the experimental design could explain the discrepancies with other studies which have addressed the issue of the FRN modulation by RPE and showed either no modulation, or modulation by either negative, or positive only RPE [for review see Walsh and Anderson (2011); San Martin (2012)]. Indeed, previous studies report that aMCC/ACC neuronal responses are sensitive to experimental context (Procyk et al., 2000; Quilodran et al., 2008; Rothe et al., 2011). In contrast to most of the gambling tasks that have been used previously, our task involved learning from both negative and positive feedback to allow an appropriate exploration of the 5 possible actions. The neuronal dissociation of responses to positive and negative feedback by aMCC cells (Ito et al., 2003; Amiez et al., 2005; Sallet et al., 2007; Quilodran et al., 2008) provide the neuronal substrates for the signed RPE modulation we observed in this experiment and in a previous fMRI experiment in humans using a similar protocol (Amiez et al., 2012). The heterogeneity of the neuronal populations coding for outcomes in the anterior part of the cingulate cortex (Ito et al., 2003; Amiez et al., 2005; Sallet et al., 2007; Quilodran et al., 2008; Kvitsiani et al., 2013) and the dopaminergic nuclei that are projecting to the prefrontal cortex (Bromberg-Martin et al., 2010) might provide the neuronal basis for a flexible system that is capable of adapting its responses depending on task-specific requirements. Performance adjustment has been correlated to FRN amplitude in some studies that required subjects to learn about reward contingencies (Cohen et al., 2007; Bellebaum and Daum, 2008; Van de Vijver et al., 2011; San Martin, 2012; Van Der Helden and Boksem, 2012; Walsh and Anderson, 2012). In the current study, the structure of the task was explained to the subjects prior to the experiment. A modulation of the FRN was then observed in conjunction with adapted behaviors. The subjects did the tasks without making almost any perseverative error, and as suggested by RTs, their expectations in obtaining the correct feedback increased with the fewer targets they had to choose among.

### Is FRN specific to feedback?

Finally we observed a FRN-like potential following a SC cue. Cingulate cells in monkeys encode events that signal the need to adapt behavior, even if the event is not a reward or an error (Amiez et al., 2005). Here we demonstrate that an FRN-like potential could be elicited by an event that is not related to action performance (the SC cue), but being nevertheless important to behavioral adaptation. In contrast with the FRN modulation by RPE for feedback, we did not observe an RPE modulation at the SC. One might have expected a modulation by the value of information rather than by the value of reward at the time of the SC. Bromberg-Martin and Hikosaka (2011) have shown that midbrain neurons can code both a RPE and information prediction error (IPE). However, the IPE value for the different SCs is constant in our task (the probability of the reward following all SC is *p* = 0.2). This fact could explain the lack of modulation of the FRN at SC. Further experiments should be conducted, specifically addressing the issue of FRN sensitivity to IPE.

The presence of an FRN-like potential whenever adaptation is required (IC1-4, C1-5, or SC0-2) suggests that the FRN participates in signaling the need for behavioral adaptation. This result is in line with recent research suggesting a general role of the a MCC in behavioral adaptation (Quilodran et al., 2008; Hayden et al., 2011b; Karlsson et al., 2012; Kolling et al., 2012). The production of a FRN after various events would suggest that the FRN source is responding to various types of adaptations. For instance an IC1-4 feedback is followed by selection of a new target on the next trial, but C1-5 and SC0-2 feedbacks are indicating the need to change strategy, i.e., switching from exploring to exploiting and from exploiting to exploring, respectively. Further analysis using time-frequency decomposition could offer new perspectives on how different types of adaption are implemented (Womelsdorf et al., 2010; Cohen et al., 2011; Rothe et al., 2011; Van de Vijver et al., 2011; Cavanagh et al., 2012; Hajihosseini and Holroyd, 2013).

## Conclusion

The present study demonstrates that the FRN is linked to the evaluation of positive and negative feedback during exploration. Such evaluation is likely to carry information necessary for appropriate adaptation, such as maintaining exploration after errors, shifting toward exploitation following the occurrence of the first positive feedback, or shifting back to exploration following the presentation of the SC. The modulation of the FRN by RPE values illustrates that the FRN also reflects a mechanism of reinforcement-based evaluation of feedback in the exploratory period. Results of the aMCC computations could lead to a regulation of decision processes in other structures, for instance in the dorsolateral prefrontal cortex or in the ventral striatum (Rothe et al., 2011; Kolling et al., 2012; Khamassi et al., 2013).

### Conflict of interest statement

The authors declare that the research was conducted in the absence of any commercial or financial relationships that could be construed as a potential conflict of interest.
